# Are pretreatment neutrophil-lymphocyte ratio and platelet-lymphocyte ratio useful in predicting the outcomes of patients with small-cell lung cancer?

**DOI:** 10.18632/oncotarget.16553

**Published:** 2017-03-24

**Authors:** Min Deng, Xuelei Ma, Xiao Liang, Chenjing Zhu, Manni Wang

**Affiliations:** ^1^ Cancer Center, State Key Laboratory of Biotherapy, West China Hospital of Sichuan University, Chengdu, Sichuan, PR China; ^2^ Department of Oncology, West China Hospital of Sichuan University, Chengdu, Sichuan, PR China

**Keywords:** small cell lung cancer (SCLC), prognostic factors, neutrophil-lymphocyte ratio (NLR), platelet-lymphocyte ratio (PLR), lactate dehydrogenase (LDH)

## Abstract

**Objectives:**

The neutrophil-lymphocyte ratio (NLR) and the platelet-lymphocyte ratio (PLR) have been proved to affect the prognosis of various types of cancers. However, the prognostic role of NLR and PLR in patients with small-cell lung cancer (SCLC) remains controversial. The objective of this study is to assess the prognostic values of NLR, PLR and other potential prognostic indexes in SCLC patients.

**Results:**

The optimal cutoff levels were 2.65 for NLR, 125 for PLR and 210 for LDH by ROC curves analysis. Patients in the NLR ≥ 2.65 and LDH ≥ 210 groups were significantly correlated with worse PFS and OS. However, patients in the PLR < 125 group presented longer PFS time than patients in the PLR ≥ 125 group. Multivariate analysis showed that NLR ≥ 2.65 was an independent risk factor for both PFS (HR = 1.38; 95% CI 1.04–1.83; *P* = 0.027) and OS (HR = 1.35; 95% CI 1.02–1.79; *P* = 0.039). LDH and the clinical stage were independent prognostic factors for PFS in SCLC patients. LDH, surgery history, thoracic RT and PCI were independent prognostic factors for OS.

**Materials and Methods:**

320 patients with SCLC were enrolled in this research from 2007 to 2014. Data was acquired through patients’ medical records and follow-ups. Receiver operating curve (ROC) was used to determine the optimal cut-off levels of NLR, PLR and lactate dehydrogenase (LDH). The Kaplan-Meier univariate analysis and multivariate Cox regression analysis were used to evaluate the impact of the NLR, PLR and other potential prognostic factors on overall survival (OS) and progressive-free survival (PFS).

**Conclusions:**

Pretreatment elevated NLR and LDH were independent factors for poor prognosis in SCLC patients. High PLR was associated with poor PFS, but it was not an independent prognostic factor for PFS and OS.

## INTRODUCTION

Lung cancer is a serious health problem with a current 5-year relative survival of 18% [1]. In recent decades, molecular-targeted therapies have been developed and have provided significant benefits for non-small cell lung cancer (NSCLC) patients in prolonging their OS [2]. However, there has been a distinct paucity of breakthroughs in the treatment of SCLC [3]. The median and 5-year survival rates have not significantly improved over the past 15 years for patients with SCLC [4]. Therefore, it is essential for us to explore simple and accessible prognostic factors for SCLC.

Emerging evidences have confirmed that the inflammatory response plays a crucial role in tumor progression. NLR is known as a systemic inflammatory marker which is calculated by dividing the circulating neutrophil counts by the lymphocyte counts. It has been proven as prognostic factors in many types of cancers, including metastatic melanoma [5], oesophageal cancer [6], colorectal cancer [7], pancreatic cancer [8], metastatic castration-resistant prostate cancer(mCRPC) [9], diffuse large B-cell lymphoma (DLBCL) [10] and NSCLC [11]. PLR is another index of systemic inflammation which is calculated by dividing the circulating platelet counts by the lymphocyte counts. Previous studies have demonstrated the prognostic role of PLR in many malignant tumors, such as breast cancer [12], nasopharyngeal cancer [13] and NSCLC [14]. However, the prognostic role of factors mentioned above in patients with small cell lung cancer (SCLC) remains controversial.

In order to evaluate the prognostic roles of NLR, PLR and other potential prognostic factors in SCLC patients with progression-free survival (PFS) and overall survival (OS), we retrospectively analyzed a large sample of patients with SCLC in this study.

## RESULTS

### The optimal cutoff levels for elevated NLR, PLR and LDH

**Figure 1:**
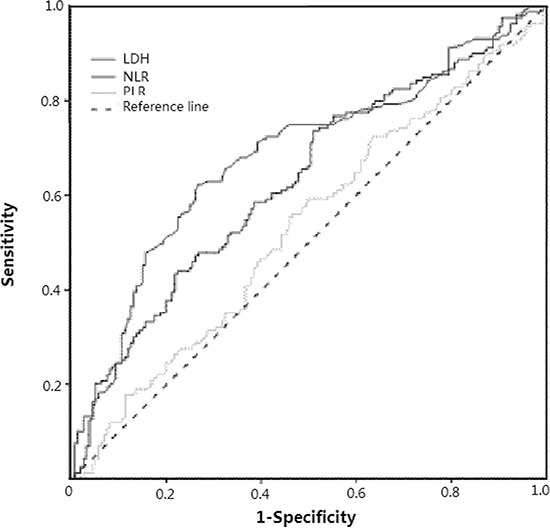
Receiver operating characteristic (ROC) curves of pretreatment NLR, PLR and LDH for predicting survival in patients with SCLC after treatments

In order to avoid a predetermined cutoff point, we used receiver operating curve (ROC) analysis to determine the optimal cutoff values of pretreatment NLR, PLR and LDH according to the maximum joint specificity and sensitivity. According to the ROC curve showed in Figure 1, the area under the ROC curves for NLR, PLR and LDH was 0.632 (95% CI: 0.571–0.693, *P* < 0.001), 0.531 (95% CI: 0.468–0.595, *P* = 0.332) and 0.683 (95% CI: 0.623–0.742, *P <* 0.001). The optimal cutoff levels were 2.65 for NLR, 125 for PLR and 210 for LDH by ROC curves analysis.

### Basic characteristics of patients

The median age of all patients was 58 (range: 24–81). The majority of the patients were male (74.7%, 239/320), and female patients account for 25.3% (81/320). Almost 61.9% (198/320) of patients were at extensive stage (ES), and 38.1% (122/320) of patients were in limited stage (LS). The ECOG performance status of patients is almost normal (median 1). Current or ever-smokers consisted 67.2% (215/320) of patients. 27 patients (8.4%) were treated with thoracic surgery, 135 patients (42.2%) were treated with thoracic radiotherapy (RT) and 77 patients (24.1%) received prophylactic cranial irradiation (PCI). The median value of NLR was 3.04 (range: 0.66–15.50). 123 patients (38.4%) were in NLR < 2.65 group and 197 patients (61.6%) were in NLR ≥ 2.65 group. The median value of PLR was 125.10 (range: 39.51–589.06), 159 patients (49.7%) were in PLR < 125 group and 161 patients (50.3%) were in PLR ≥ 125 group. The median value of LDH was 205 (range: 104–1887). 169 patients (52.8%) were in LDH < 210 group and 151 patients (47.2%) were in LDH ≥ 210 group. The median PFS and median OS of all the patients were 7.65 months (range: 0–80) and 13.8 months (range: 0.6–80, respectively). The correlation between NLR, PLR and clinical factors of SCLC patients were shown in Table 1. Our study revealed that NLR was prominently associated with tumor stage, PS, LDH, surgery, and RT. PLR was closely associated with the gender, history of tobacco, LDH and RT, separately.

**Table 1:** Correlation between peripheral NLR/PLR and clinical variables of SCLC patients

Variables	cases	NLR		*P*	PLR		*P*
		< 2.65	≥ 2.65		< 125	≥ 125	
Age (years)							
< 55	113	41	72		53	60	
≥ 55	207	82	125	0.558	106	101	0.462
gender							
male	239	90	149		129	110	
female	81	33	48	0.622	30	51	0.008
Smoking history							
no	105	47	58		43	62	
yes	215	76	139	0.104	116	99	0.029
Clinical stage							
LS	122	61	61		68	54	
ES	198	62	136	0.001	91	107	0.089
Performance status (PS)							
0	104	52	52		55	49	
≥ 1	216	71	145	0.003	104	112	0.427
LDH							
< 210	169	90	79		101	68	
≥ 210	151	33	118	< 0.001	58	93	< 0.001
surgery							
no	293	105	188		143	150	
yes	27	18	9	0.002	16	11	0.299
Thoracic radiotherapy (RT)							
no	185	59	126		82	103	
yes	135	64	71	0.005	77	58	0.025
Prophylactic cranial irradiation (PCI)							
no	243	88	155		117	126	
yes	77	35	42	0.146	42	35	0.328

### PFS and OS according to NLR, PLR and LDH status

**Figure 2:**
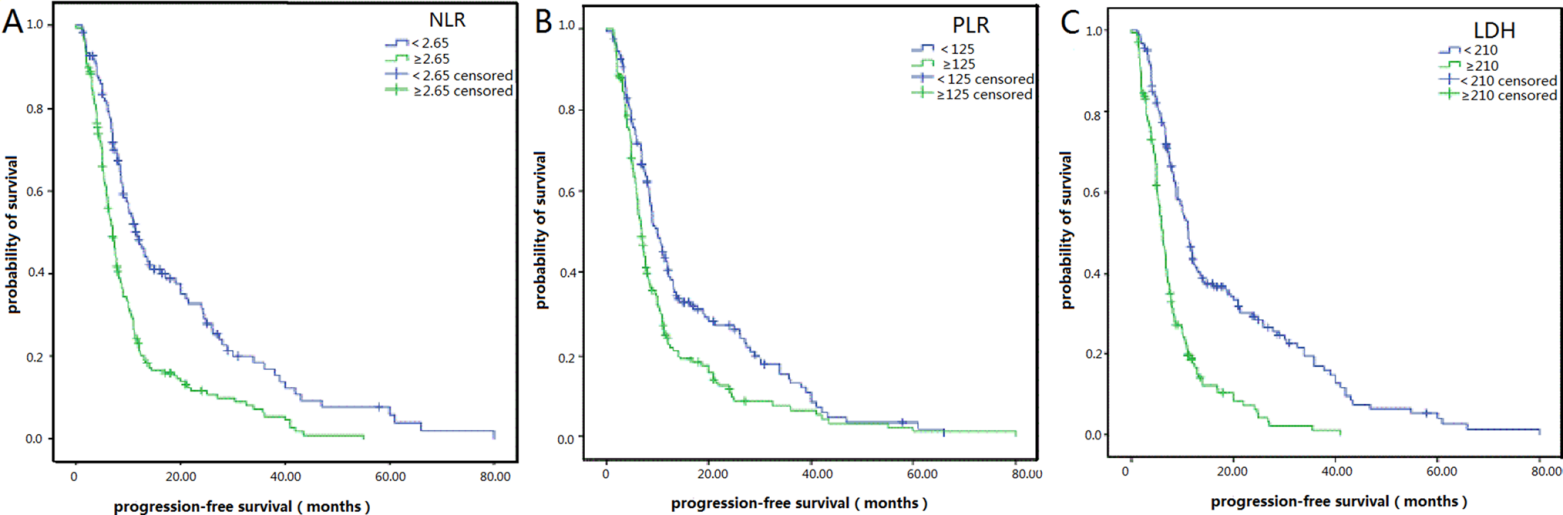
Kaplan–Meier survival curves for progression-free survival (PFS) in SCLC patients after diagnoses (**A**) PFS of patients with NLR < 2.65 was longer than those with NLR ≥ 2.65. (*P <* 0.001, log-rank). (**B**) PFS of patients with PLR < 125 was longer than those with PLR ≥ 125. (*P* = 0.001, log-rank). (**C**) PFS of patients with LDH < 210 was longer than those with LDH ≥ 210. (*P <* 0.001, log-rank).

**Figure 3:**
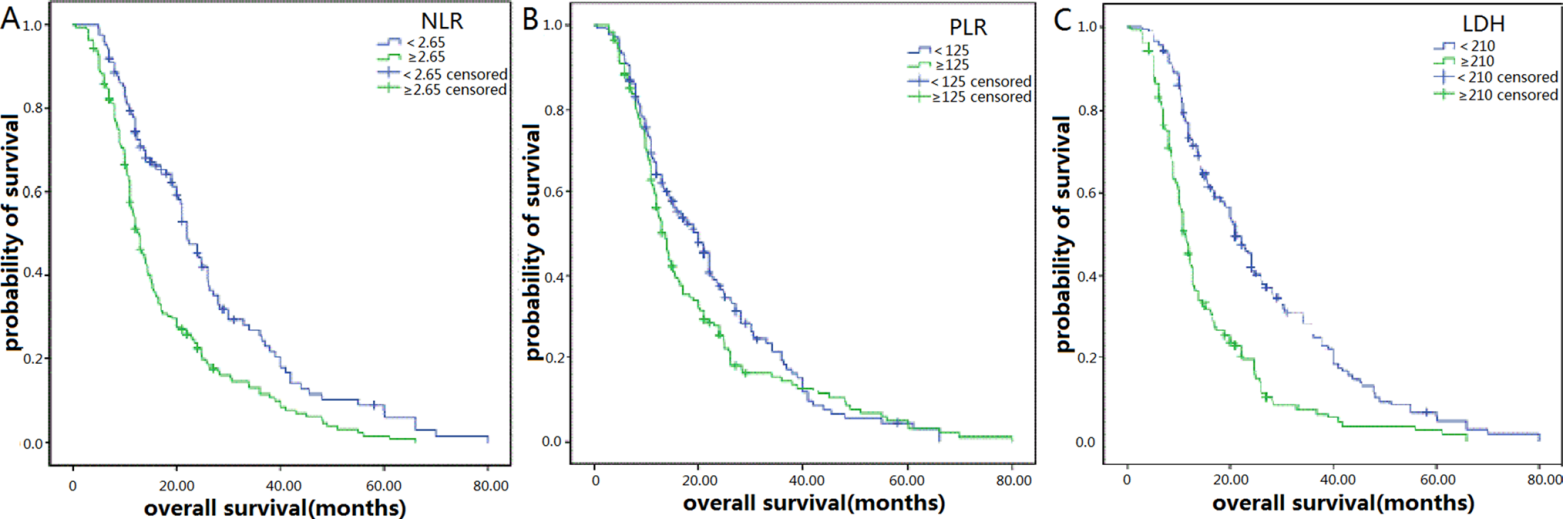
Kaplan–Meier survival curves for overall survival (OS) in patients with SCLC after diagnoses (**A**) OS of patients with NLR < 2.65 was also longer than those with NLR ≥ 2.65. (*P <* 0.001, log-rank). (**B**) OS of patients with PLR < 125 was not obvious different from those with PLR ≥ 125. (*P* = 0.099, log-rank).(**C**) OS of patients with LDH < 210 was also longer than those with LDH ≥ 210. (*P <* 0.001, log-rank).

As is shown in Table 2, in the NLR < 2.65 group, the 1-, 2-, and 3-year PFS rates separately were 41.7, 22.0 and 9.8%, while in the NLR ≥ 2.65 group, the PFS rates were 19.8, 7.7 and 3.6% (Figure 2A). In the PLR < 125 group, the 1-, 2-, and 3-year PFS rates were 35.4, 7.6 and 2.5%, and in the PLR ≥ 125 group, the PFS rates were 20.4, 8.6 and 4.3% (Figure 2B). In the LDH < 210, the 1-, 2-, and 3-year PFS rates were 40.2, 20.7 and 4.1%, while in the LDH ≥ 210 group, the PFS rates were 13.9, 4.7 and 0.7% (Figure 2C). Correspondingly, the 1-, 2-, and 3-year OS rates were 74.8, 35.8 and 17.1% in the NLR < 2.65 group and 51.3, 18.8 and 8.6% in the NLR ≥ 2.65 group, separately (Figure 3A). The 1-, 2-, and 3-year OS rates were 63.3, 27.9 and 12.7% in the PLR < 125 group and 57.4, 22.8 and 10.5% in the PLR ≥ 125 group (Figure 3B). The 1-, 2-, and 3-year OS rates were 74.0, 35.5 and 18.3% in the LDH < 210 group and 45.0, 13.9 and 4.6% in the LDH ≥ 210 group (Figure 3C). On a whole, PFS and OS of patients in the NLR < 2.65 and LDH < 210 group were obviously improved compared with patients in the NLR ≥ 2.65 and LDH ≥ 210 groups. However, only PFS of patients in the PLR < 125 group were relatively longer than patients in PLR ≥ 125 group.

**Table 2:** Prognostic factors for PFS and OS by univariate analysis

variables	*n*	PFS(year)			*p*	OS(years)			*p*
		1	2	3		1	2	3	
Age (years)									
< 55	113	31.9%	16.8%	9.7%		63.7%	32.7%	17.7%	
≥ 55	207	25.6%	11.1%	3.9%	0.022	58.5%	21.3%	8.7%	0.007
gender									
male	239	25.1%	11.7%	5.4%		55.6%	23.0%	11.3%	
female	81	35.8%	17.3%	7.4%	0.014	74.1%	32.1%	13.6%	0.043
Smoking history									
no	105	34.3%	17.1%	7.6%		70.5%	33.4%	12.4%	
yes	215	24.7%	11.1%	5.1%	0.010	55.3%	21.4%	11.6%	0.027
Clinical stage									
LS	122	45.9%	23.8%	14.8%		72.1%	35.3%	18.9%	
ES	198	16.7%	6.6%	0.5%	< 0.001	53.0%	19.2%	7.6%	< 0.001
Performance status (PS)									
0	104	33.7%	17.3%	5.8%		71.2%	32.7%	13.5%	
≥ 1	216	25.0%	11.1%	6.0%	0.017	55.1%	21.7%	11.1%	0.006
NLR									
< 2.65	123	41.7%	22.0%	9.8%		74.8%	35.8%	17.1%	
≥ 2.65	197	19.8%	7.7%	3.6%	< 0.001	51.3%	18.8%	8.6%	< 0.001
PLR									
< 125	159	35.4%	7.6%	2.5%		63.3%	27.9%	12.7%	
≥ 125	161	20.4%	8.6%	4.3%	0.001	57.4%	22.8%	10.5%	0.099
LDH									
< 210	169	40.2%	20.7%	4.1%		74.0%	35.5%	18.3%	
≥ 210	151	13.9%	4.7%	0.7%	< 0.001	45.0%	13.9%	4.6%	< 0.001
surgery									
no	293	24.9%	10.2%	3.4%		57%	22.2%	9.2%	
yes	27	59.3%	44.4%	33.3%	< 0.001	96.3%	59.2%	40.7%	0.001
Thoracic radiotherapy (RT)									
no	185	19.5%	9.2%	4.3%		51.4%	18.9%	9.7%	
yes	135	39.3%	18.5%	8.1%	< 0.001	72.6%	34.2%	14.8%	0.001
Prophylactic cranial irradiation (PCI)									
no	243	25.4%	10.2%	4.9%		57.2%	23.0%	8.6%	
yes	77	35.1%	22.1%	9.1%	0.052	70.1%	32.5%	22.1%	0.005

### Prognostic factors of SCLC patients

For all patients, PFS rates at the 1-, 2- and 3-year period respectively were 27.8, 13.1 and 5.9%. OS rates at the 1-, 2- and 3-year period respectively were 60.3, 25.3 and 11.9%. Univariate analysis showed that NLR ≥ 2.65, PLR ≥ 125, LDH ≥ 210, age ≥ 55, male, smoking, ES-SCLC, PS ≥ 1 and deletion of surgery or thoracic RT were associated with significantly shorter PFS in SCLC patients. Similarly, NLR ≥ 2.65, LDH ≥ 210, age ≥ 55, male, smoking, ES-SCLC, PS ≥ 1 and patients without surgery, thoracic RT or PCI predicted worse OS (Table 2).

The Cox proportional hazard model was applied to perform multivariate analyses. The analysis revealed that NLR ≥ 2.65 (HR = 1.38; 95% CI 1.04–1.83; *P* = 0.027), LDH ≥ 210 (HR = 1.56; 95% CI 1.18–2.07; *P* = 0.002) and ES-SCLC (HR = 1.62; 95% CI 1.18–2.23; *P* = 0.003) were independent prognostic factors for PFS in patients with SCLC. Correspondingly, NLR ≥ 2.65 (HR = 1.35; 95% CI 1.02–1.79; *P* = 0.039), LDH ≥ 210 (HR = 1.46; 95% CI 1.10–1.96; *P* = 0.002), patient with surgery (HR = 0.55; 95% CI 0.33–0.93; *P* = 0.025), thoracic RT (HR = 0.66; 95% CI 0.50–0.88; *P* = 0.005) and PCI (HR = 0.71; 95% CI 0.53–0.96; *P* = 0.023) were independent prognostic factors for OS in SCLC patients (Table 3).

**Table 3:** Prognostic factors for PFS and OS as determined by multivariate Cox proportional hazards regression model

variables	PFS		*P*	OS		*P*
	HR	95% CI		HR	95% CI	
Clinic stage	1.62	1.18–2.23	0.003			
NLR	1.38	1.04–1.83	0.027	1.35	1.02–1.79	0.039
LDH	1.56	1.18–2.07	0.002	1.46	1.10–1.96	0.010
Surgery				0.55	0.33–0.93	0.025
Thoracic RT				0.66	0.50–0.88	0.005
PCI				0.71	0.53–0.96	0.023

## DISCUSSION

Recently, many articles have certified that the progression of cancers was closely associated with inflammation and immunity status, and that the NLR, PLR and LDH were the easily obtained and effective markers of inflammation and immunity. However, currently there are no consistent cut off values in preexisting studies. Therefore, we determined the optimal cutoff values of 2.65 for NLR, 125 for PLR and 210 for LDH by ROC curves analysis.

As is known, the neutrophil, as a kind of inflammation cell, influences tumor initiation and progression in the tumor microenvironment [15]. It can aid the proliferation and survival of malignant cells, promote angiogenesis and metastasis, subvert adaptive immune responses, and alter responses to hormones and chemotherapeutic agents [16, 17]. The neutrophil also increases VEGF (vascular endothelial growth factor) bioavailability and bioactivity [18], and VEGF-mediated angiogenesis is essential for tumor growth, recurrence, invasion and metastasis [19]. Moreover, multiple clinical and experimental studies have established that the lymphocyte also plays an important role in antitumor immunity [20, 21]. The high NLR which means an increased neutrophil count or a decreased lymphocyte count indicates an imbalance in the inflammatory cascade and immune response to malignant tumors, thus tumors recurrence and metastasis may occur more frequently in the high NLR patients [18].

In our study, we found patients with elevated NLR ( ≥ 2.65) had obviously worse PFS and OS than those with low NLR (< 2.65) (Figures 2.A and 3.A, Table 2). The multivariate analysis showed NLR ≥ 2.65 was an independent prognostic factor of worse PFS and OS. The association between NLR and prognosis in SCLC patients has also been confirmed in some other studies. For example, Shao et al. [22] found out that high NLR was a useful and easily obtained indicator for recurrence which predicted a poor prognosis for C-SCLC (combined-SCLC). Wang et al. [23] had the same opinion. Hong et al. [24] showed that high NLR predicted poor long-term prognosis in univariate analysis, but multivariate analysis showed NLR was not an independent prognostic factor in SCLC patients. However, Kang et al. [25] demonstrated that NLR was an independent prognostic factor for OS and PFS, which was consistent with our opinion.

PLR is another indicator of systemic inflammation, and some studies have demonstrated that the activation of platelets and the coagulation system are crucial to tumor metastasis. But the potential mechanisms have not been well clarified until now, some scholars hold the opinion that platelet provides a procoagulant surface to promote the enlargement of cancer-related coagulation, which can be used to shroud tumor cells, thus shielding the tumor cells from immune responses and facilitating them to grow and metastasis [26, 27]. Another opinion is that platelets are the main sources of cytokines such as VEGF and transforming growth factor β (TGF-β) which have significant effect on tumor angiogenesis [28]. In a word, the high PLR means an increased platelet count or a decreased lymphocyte count, which may be associated with tumor recurrence and metastasis.

Our study showed patients with elevated PLR ( ≥ 125) had significantly worse PFS than those with low PLR (<125) (Figure 2B, Table 2), but was not associated with OS in SCLC patients (Figure 3B, Table 2), which was consistent with the finding of Zhao et al. [14] and Kang et al. [25]. The multivariate analysis showed that PLR ≥ 125 was not an independent prognostic factor of worse PFS and OS in SCLC patients.

Earlier studies have confirmed LDH was a strong, independent predictive factor of survival in patients with SCLC [29, 30, 31]. In our study, we also found patients with elevated LDH ( ≥ 210) had obviously worse PFS and OS than those with low NLR (< 210) (Figures 2C and 3C, Table 2). The multivariate analysis showed LDH ≥ 210 was an independent prognostic factor of worse PFS and OS.

## MATERIALS AND METHODS

### Patients

In this retrospective study, we analyzed 320 patients with pathologically confirmed SCLC in our cancer center from March 2007 to December 2014. The median follow-up time was 39.1 months (range: 3.2–85.4). From electronic records, we extracted patient characteristics including sex, age, smoking habit, Eastern Cooperative Oncology Group (ECOG) performance status, diagnoses, treatments, outcomes and the blood results at the time of diagnosis. The primary endpoint was PFS, which was calculated from the date of first diagnosis to the onset of disease progression or the last follow-up. The second endpoint was OS, defined as the period from diagnosis to the time of death or the last follow-up.

### Statistical analysis

We used SPSS statistical software (version 19.0) to analyze data, and the chi-square test to compare categorical variables. Kaplan-Meier method was used for univariate analysis. Obvious differences between groups were determined by log-rank test. The Cox proportional hazard model was applied for multivariate analysis, and hazard ratios (HRs) obtained were reported as relative risks with corresponding 95% confidence intervals (CIs). All tests were 2-sided, and *p value* of < 0.05 was considered statistical significant.

## CONCLUSIONS

Our study observed that elevated peripheral NLR before treatment was an independent prognostic factor of poor PFS and OS in SCLC patients. Patients in elevated PLR group had relatively shorter PFS time than those in low PLR group, but PLR was not an independent prognostic indicator of poor PFS and OS. We also found LDH and clinical stage were independent prognostic factors for PFS. Correspondingly, LDH, surgery history, thoracic RT and PCI were independent prognostic factors for OS in SCLC patients. Those findings might help clinicians to use personalized and reasonable adjuvant therapies for the patients with high risk of recurrence and metastasis after diagnose. We should note that this study is limited for it is a single-center and retrospective research. In the future, multicenter and prospective studies are needed to testify our findings.
